# Pervasive genotype-by-environment interactions shape the fitness effects of antibiotic resistance mutations

**DOI:** 10.1098/rspb.2023.1030

**Published:** 2023-08-30

**Authors:** Jake K. Soley, Matthew Jago, Calum J. Walsh, Zahra Khomarbaghi, Benjamin P. Howden, Mato Lagator

**Affiliations:** ^1^ Division of Evolution and Genomic Sciences, School of Biological Sciences, Faculty of Biology, Medicine and Health, University of Manchester, Manchester M13 9PL, UK; ^2^ Department of Microbiology and Immunology, University of Melbourne, at the Peter Doherty Institute for Infection and Immunity, Melbourne, Victoria 3000, Australia; ^3^ Centre for Pathogen Genomics, University of Melbourne, Melbourne, Victoria 3000, Australia

**Keywords:** antibiotic resistance, experimental evolution, genotype-by-environment, rifampicin

## Abstract

The fitness effects of antibiotic resistance mutations are a major driver of resistance evolution. While the nutrient environment affects bacterial fitness, experimental studies of resistance typically measure fitness of mutants in a single environment only. We explored how the nutrient environment affected the fitness effects of rifampicin-resistant *rpoB* mutations in *Escherichia coli* under several conditions critical for the emergence and spread of resistance—the presence of primary or secondary antibiotic, or the absence of any antibiotic. Pervasive genotype-by-environment (GxE) interactions determined fitness in all experimental conditions, with rank order of fitness in the presence and absence of antibiotics being strongly dependent on the nutrient environment. GxE interactions also affected the magnitude and direction of collateral effects of secondary antibiotics, in some cases so drastically that a mutant that was highly sensitive in one nutrient environment exhibited cross-resistance to the same antibiotic in another. It is likely that the mutant-specific impact of *rpoB* mutations on the global transcriptome underpins the observed GxE interactions. The pervasive, mutant-specific GxE interactions highlight the importance of doing what is rarely done when studying the evolution and spread of resistance in experimental and clinical work: assessing fitness of antibiotic-resistant mutants across a range of relevant environments.

## 1. Introduction

The rise of antimicrobial resistance in recent decades represents an immense global health and economic risk, particularly in developing nations, with stark estimates of the cost to human life [1]. Efforts to combat the rise of resistance have largely focused on the development of new drugs. Yet, while the last century enjoyed unprecedented levels of new drug discovery, the pipeline has dried up in recent years [2]. The dramatic slowdown in the development of new drugs has resulted in the need for novel approaches to tackle resistance as well as preserving the usefulness of existing drugs [3]. Improving our understanding of the evolutionary processes underpinning resistance is essential for prolonging the lifetime of existing drugs and, hence, essential for slowing down the spread of antimicrobial resistance. This includes understanding the factors that impact how resistance emerges, what effect mutations have on phenotype and fitness, and how they influence the evolutionary fate of a population.

Bacteria can evolve resistance through spontaneous DNA mutations, often single-point mutations, that inhibit drug binding or action [4]. While in many cases there are multiple possible mutations that can confer resistance to an antibiotic, not all mutations confer the same level of resistance. Therefore, some mutants will have lower fitness than others in the presence of an antibiotic (sometimes referred to as ‘survival’ or just ‘resistance’) [5]. Some resistance mutations also impose *collateral effects*, by altering fitness in the presence of secondary antibiotics to which the population has not been previously exposed [6,7]. Furthermore, resistance mutations are often associated with a reduction in fitness in an antibiotic-free environment (commonly referred to as the *fitness cost of resistance*) [8], which can arise as a consequence of the resistance mutation altering the structure or function of an essential protein [9,10]. For example, resistance to rifampicin often carries a fitness cost as the resistance-conferring mutations occur in the *rpoB* gene, which encodes the *β*-subunit of RNA polymerase [10,11]. The likelihood of acquiring and maintaining multi-drug resistance critically depends on the fitness effects of resistance mutations in the presence and absence of the primary antibiotic, as well as the associated collateral effects in the presence of secondary antibiotics [12–14].

The fitness effects of mutations, therefore, depend not only on the genotype but also on the environment [15,16]. Broadly speaking, when attempting to understand the key factors that affect the emergence and spread of drug resistance, it is critical to characterize fitness in several environments: (i) the presence of the primary antibiotic to which resistance has originally evolved; (ii) the absence of any antibiotic, as would occur when the primary treatment is withdrawn and as measured by the fitness cost of resistance; (iii) the presence of a secondary antibiotic used to treat the resistant infection, captured by the collateral effects; and (iv) low antibiotic concentrations, as concentrations vary drastically between different niches in the body and the environment [17,18]. The fitness associated with a mutation can vary considerably between these environments [15,19].

However, the presence and absence of an antibiotic is not the only environmental factor that determines the fitness effect of a mutation. The fitness effects of a resistance mutation can depend on the nutrient composition of the environment, resulting in a genotype-by-environment (GxE) interaction [15,16]. The existence of such GxE interactions means that, at a given concentration of an antibiotic, one mutant might outperform another in one nutrient environment, while exhibiting lower fitness in a different environment [20]. GxE interactions are difficult to predict, especially when mutations have pleiotropic effects that affect many phenotypes [10,21–23]. The complexity of environments in which bacteria live means that the effect of GxE interactions on fitness can play a key role in determining the emergence and spread of antimicrobial resistance. And yet, the majority of studies measuring fitness of resistance mutations tend to do so in a single nutrient environment, only varying whether an antibiotic is present or not [23–25].

We investigated how GxE interactions (in this study, we consider the environment to be the nutrient composition of the growth media) affect bacterial fitness in the presence and absence of the primary antibiotic, as well as the collateral effects of secondary antibiotics. We focused on 11 genetically identical mutants that differ only with respect to a single-point mutation in the *rpoB* gene (coding for the β-subunit of RNA polymerase), which confers resistance to the antibiotic rifampicin. We identified complex GxE interactions that make fitness effects of resistance mutations and the collateral effects of other antibiotics strongly dependent on the nutrient environment.

## 2. Results

### (a) GxE interactions alter rank order of fitness in the primary antibiotic environment

**Figure 1.**
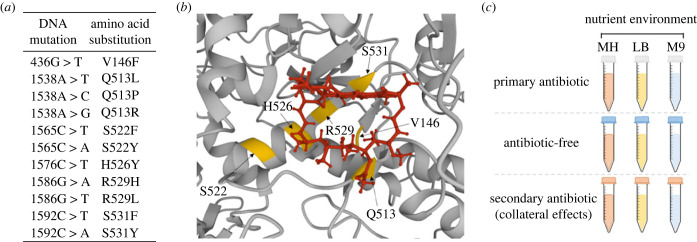
Investigating genotype-by-environment interactions of *rpoB* mutations. (*a*) *rpoB* mutants (and the respective amino acid substitutions) generated in this study. (*b*) Location of the affected residues (yellow) and their proximity to a rifampicin molecule (red) in the three-dimensional structure of RNA polymerase (grey; structure PDB ID: 4KMU) [26–28]. (*c*) Schematic outlining the environments investigated herein. In this instance the primary antibiotic was rifampicin, and the secondary antibiotic was chloramphenicol, ciprofloxacin, d-cycloserine, streptomycin, or tetracycline. MH, Mueller-Hinton media; LB, lysogeny broth; M9, M9 media supplemented with 2% glucose.

One of the key factors that determine the evolutionary success of a newly acquired resistance mutation is its fitness effects in the presence of the selective (primary) antibiotic. To explore the relationship between nutrient environment and fitness in the presence of the primary antibiotic, we generated 11 rifampicin-resistant strains of *Escherichia coli* using a fluctuation assay-based approach (see Materials and methods). Each strain was genetically identical to the wildtype (WT) (BW25113, carrying a *tolC* deletion to prevent the activity of TolC-dependent efflux pumps) with the exception of a single substitution mutation in *rpoB* (figure 1*a*). All these mutations (except V146F) are within the rifampicin resistance determining region of the *rpoB* gene, and all (including V146F) lie close to the rifampicin binding site (figure 1*b*). We measured fitness (as the area under the curve (AUC) of growth curves; electronic supplementary material, figure S1) of all 12 strains (11 *rpoB* mutants and the WT) in three common laboratory nutrient environments: Mueller-Hinton (MH) broth, lysogeny broth (LB) and M9 minimal media with 2% glucose (M9), in the presence of 8 µg ml^−1^ of the primary antibiotic rifampicin (figure 1*c*). This concentration of rifampicin was selected as we determined it to be the minimum inhibitory concentration (MIC) of the WT strain measured in MH. We used AUC as a measure of fitness as it is a well-used growth metric that integrates various features of the bacterial growth curve, such as growth rate and lag phase [29–32].

**Figure 2.**
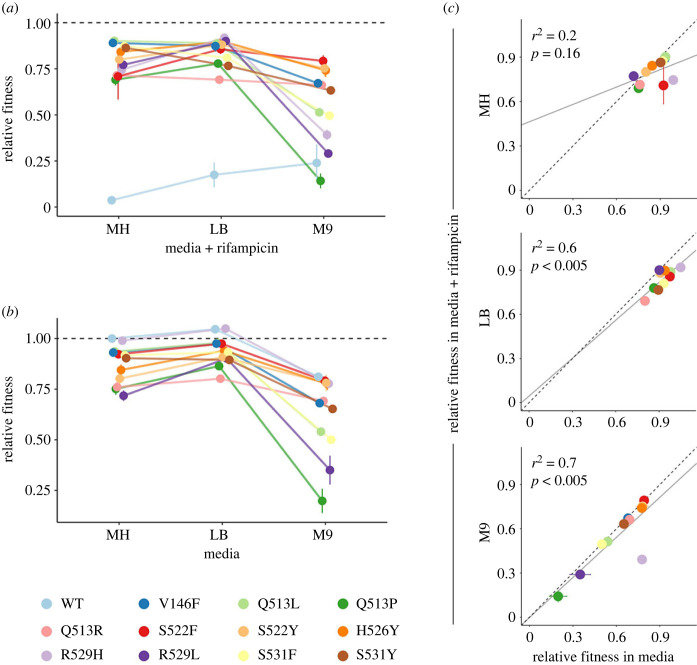
GxE interactions of *rpoB* mutations influence fitness in the presence and absence of rifampicin. (*a*) Relative fitness of mutants and the WT grown in three nutrient environments supplemented with 8 µg ml^−1^ rifampicin (determined as the MIC of the WT in MH media; whilst some growth of the WT was observed in LB and M9 with this concentration, it was maintained in all three environments for consistency). (*b*) Relative fitness of mutants and the WT grown in three antibiotic-free nutrient environments. (*c*) Relative fitness of mutants in each antibiotic-free nutrient environment against relative fitness in media supplemented with rifampicin. Results of linear regression analysis are shown in grey (solid line); dotted line shows perfect correlation. WT was excluded from regression analysis as its fitness was unsurprisingly lower in the presence of rifampicin. Points in all plots show mean of seven biological replicates; error bars show standard error of the mean. Relative fitness values are AUC values normalized to the AUC of the WT in antibiotic-free MH.

The rank order of all mutants varied considerably between environments, indicating that the genotypes interact significantly with nutrient environment (aligned ranks transformation ANOVA: *F*_20,198_ = 27.93, *p* < 0.0001) (figure 2*a*). The effect of nutrient environment can be so dominant that one mutant (Q513P) exhibited significant levels of resistance in MH and LB but showed very little growth in M9. Such prominent GxE interactions, which alter the rank order of mutant fitness in the presence of rifampicin between environments, can result in one mutant (e.g. R529H) having the highest fitness in one environment (LB), but exhibiting low relative fitness in another (M9).

### (b) GxE interactions alter rank order of fitness in the absence of antibiotics

Once a bacterial infection has evolved resistance to the initial treatment, the use of that first antibiotic will likely be withdrawn [33]. Strains carrying primary resistance mutations may then occupy an antibiotic-free environment as the drug is cleared from the body. To explore GxE interactions in the absence of antibiotics, we measured fitness of all 12 strains in the 3 nutrient environments. Around half of the mutants exhibited a significant fitness cost compared to the WT (MH: 5; LB: 5; M9: 6; for all pairwise comparisons see electronic supplementary material). No mutant had higher fitness than the WT in the absence of antibiotics. Interestingly, we observed greater diversity of fitness amongst *rpoB* mutants when grown in M9 than when tested in MH or LB media, in both the presence and absence of the primary antibiotic (standard deviations—MH: 172.5; LB: 124.3; M9: 337.3; MH + Rif: 211.3; LB + Rif: 144.5; M9 + Rif: 345.5) (figure 2). Why the variation in fitness between *rpoB* mutants might be more constrained in some environments than in others is unclear.

GxE interactions affected the rank order of mutant fitness between nutrient environments in the absence of antibiotics (aligned ranks transformation ANOVA: *F*_22,216_ = 23.42, *p* < 0.0001) (figure 2*b*). The observation that the rank order of fitness depends on the nutrient environment suggests that the maintenance of resistance mutations is contingent on both the specific mutation and the growth conditions. For example, consider the mutants Q513R and S531F. In MH media, the fitness of Q513R (mean = 1251.9, s.e.m. = 43.4) was significantly lower than the fitness of S531F (mean = 1510, s.e.m. = 17.5) (Tukey pairwise comparison: *p* < 0.005), suggesting that if they were to coexist in a population S531F would outcompete Q513R. However, in M9 the opposite was true (Q513R: mean = 1138.5, s.e.m. = 11.03; S531F: mean = 823.6, s.e.m. = 31.6; Tukey pairwise comparison: *p* < 0.0001), suggesting that in different nutrient environments the population dynamics of the primary resistant mutants are shifted. The ability to predict the long-term evolutionary success of these two mutants is further complicated by the fact that their rank order of fitness in the presence of the primary antibiotic rifampicin is also dependent on the nutrient environment (figure 2*a*).

### (c) Nutrient environment alters the relationship between fitness in the presence and absence of rifampicin

In some instances, the fitness of resistant mutants in the presence of the primary antibiotic is correlated with the fitness in its absence [34,35]*.* To determine whether fitness in the absence of rifampicin was a significant predictor of fitness in the presence of the drug, we fitted a linear regression model on the mean fitness of 11 mutants, in each of the 3 environments (the WT was excluded from the analysis as its fitness in rifampicin is unsurprisingly diminished—see Materials and methods). In LB and M9, the fitness of *rpoB* mutants in the absence and presence of rifampicin was positively correlated, while no such relationship was identified in MH (figure 2*c*). Therefore, fitness in the presence of rifampicin can be predicted based on mutant fitness in the absence of the drug only under some nutrient conditions.

Interestingly, whether the addition of rifampicin significantly altered mutant fitness was also environment dependent, as we identified a significant interaction between genotype, nutrient environment and the presence/absence of rifampicin (mixed effect model: *F*_20,393_ = 2.77, *p* < 0.001). This means that, for example, the presence of rifampicin did not significantly alter the fitness of the mutant S531F in M9 (Welch two sample *t*-test: *t*_11.06_ = 0.2, *p* = 0.85), while in MH and LB its fitness was significantly different in the presence and absence of rifampicin (MH: *t*_11.95_ = 2.85, *p* < 0.05; LB: *t*_9.38_ = 7.83, *p* < 0.001). The existence of a relationship between fitness in the absence and presence of antibiotic in two of the three media environments further demonstrates the complex GxE interactions that shape the evolutionary fate of rifampicin resistance mutations.

### (d) GxE interactions shape collateral effects of secondary antibiotics

The fitness effects of a resistance mutation in the presence of the primary antibiotic critically determine the likelihood of that mutation being fixed during treatment, while its persistence in the population following treatment is to a larger extent determined by its fitness in the absence of antibiotics. We have shown that GxE interactions play a large role in determining the fitness effects of mutations in both conditions. However, when considering the emergence of multi-drug resistance, another factor plays a major role: the collateral effects of resistance mutations—i.e. their fitness effects in the presence of a secondary antibiotic [12,36]. To test if GxE interactions shape collateral effects, we measured the MIC of all 11 mutants to 5 secondary antibiotics with differing mechanisms of action from rifampicin (chloramphenicol, ciprofloxacin, d-cycloserine, streptomycin and tetracycline) in the 3 nutrient environments. This includes drugs that have been trialled in combinatorial therapy with rifampicin [37,38], as a treatment for rifampicin-resistant tuberculosis [39], or in the case of ciprofloxacin, have a similar mode of action to the anti-tubercular drug moxifloxacin [40].

**Figure 3.**
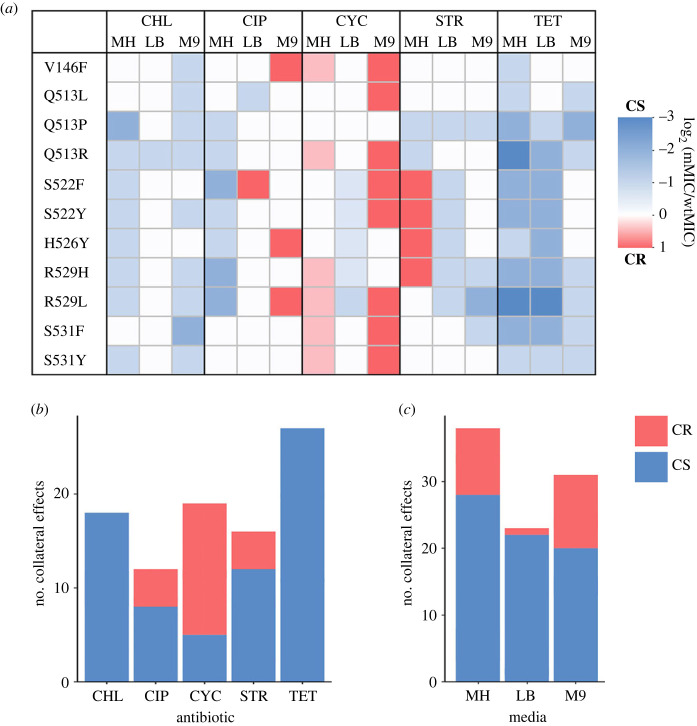
Pervasive collateral effects of *rpoB* mutants are genotype- and environment-dependent. (*a*) Collateral sensitivity (CS; blue) and cross-resistance (CR; red) of *rpoB* mutants against five secondary antibiotics (CHL, chloramphenicol; CIP, ciprofloxacin; CYC, d-cycloserine; STR, streptomycin; TET, tetracycline), in three nutrient environments. Strength of collateral effects are determined as the log base 2 of the MIC of the mutant divided by the MIC of the WT in each respective nutrient environment/antibiotic combination. (*b*) Total number and direction of collateral effects against each secondary antibiotic, and (*c*) in each nutrient environment.

Collateral effects were very common in rifampicin-resistant mutants, with mutants having different MIC compared to the WT strain in 55% of the tested environment/antibiotic combinations (figure 3). Interestingly, even mutations at the same residue but with a different amino acid substitution exhibited different collateral effects. For example, a substitution to leucine at position 513 (Q513L) did not increase the sensitivity to streptomycin, while the proline substitution did (Q513P). Whether the collateral effects were more likely to result in increased sensitivity (lower MIC than WT) or cross-resistance (higher MIC than WT) depended on the antibiotic and the mutant (figure 3*a*,*b*). For example, while cross-resistance was the most common collateral effect observed against d-cycloserine (14/19), no mutants had cross-resistance to tetracycline or chloramphenicol, instead exhibiting only varying degrees of collateral sensitivity. The likelihood of observing collateral effects also varied between antibiotics (figure 3*b*).

In addition to the antibiotic and the mutant itself, the nutrient environment also had an impact on collateral effects (figure 3*a*,*c*). This genotype-by-environment-by-antibiotic interaction means that the strength of cross-resistance or sensitivity, and in some cases even the direction of the collateral effect, is dependent not only on which secondary antibiotic is used but also on the nutrient environment. One of the more dramatic examples is S522F, which exhibited cross-resistance to ciprofloxacin in LB but increased sensitivity in MH. The same mutant exhibited cross-resistance to streptomycin in MH but collateral sensitivity in LB. The nutrient environment also impacted the total number of cross-resistance interactions—in LB only 1 such interaction was found (S522F against ciprofloxacin), while in M9 there were 11 examples of cross-resistance (figure 3*c*). Meanwhile, mutants grown in MH exhibited the highest number of collateral sensitivity interactions—28, whilst in M9 only 20 such interactions were observed. These findings demonstrate the environment-dependent variability in not only the strength but also the direction of collateral effects.

### (e) Genotype-by-environment-by-antibiotic interactions shape fitness at sub-MICs of secondary antibiotics

Differences in collateral effects (figure 3) identified certain concentrations of secondary antibiotics that are permissive to the growth of some mutants, but not others. GxE effects on collateral sensitivity ensure that the same concentration of antibiotic might not be sufficient to control the bacterial population as the nutrient environment changes. Another level of complexity arises from the fact that concentrations of antibiotics vary dramatically during the course of treatment [41], potentially leaving bacteria in the presence of sub-inhibitory concentrations (sub-MICs) of secondary antibiotics. This led us to question whether the differences in collateral effects (figure 3) correlate with fitness at low concentrations of secondary antibiotics, thus influencing the evolution of mutants when the concentration is below the MIC. Is the fitness of rifampicin-resistant mutants in the presence of sub-MICs of secondary antibiotics similarly influenced by GxE interactions?

To explore these questions, we measured the fitness of the mutants in all three nutrient environments in the presence of sub-MICs of three antibiotics—ciprofloxacin, d-cycloserine and tetracycline. We selected half the concentration of the lowest MIC of any mutant (measured in each nutrient environment separately) as our sub-MIC.

**Figure 4.**
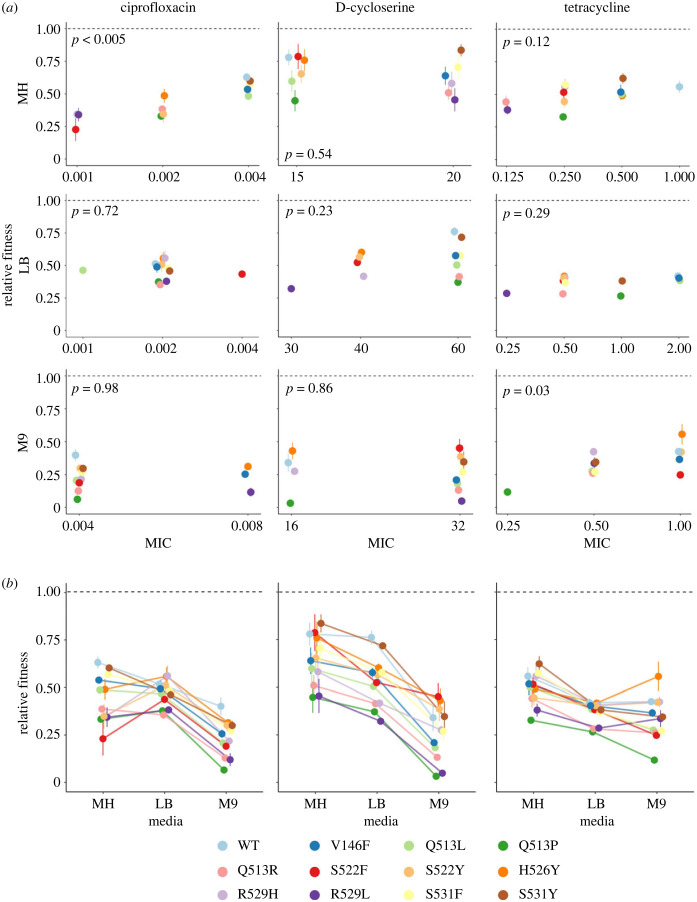
GxE interactions shape fitness of *rpoB* mutants at sub-MICs of secondary antibiotics. (*a*) Relative fitness of mutants grown at sub-MICs of secondary antibiotics ciprofloxacin (left), d-cycloserine (middle) and tetracycline (right), in the three nutrient environments, plotted against the MIC of each mutant in the corresponding nutrient environment–antibiotic combination. *p*-values from a linear mixed-effects model are shown in grey. (*b*) Relative fitness of mutants in sub-MICs of secondary antibiotics in each nutrient environment, plotted to highlight GxE interactions. Points are mean of 4 biological replicates; error bars are standard error of the mean. Fitness values are AUC values normalized to the AUC of the WT in antibiotic-free MH.

We found inconsistent and unpredictable fitness of *rpoB* mutants in sub-MIC doses of secondary antibiotics (figure 4*a*; electronic supplementary material, figure S3). Contrary to our hypothesis, we identified a significant relationship between MIC and fitness in sub-MIC of the same antibiotic in only two tested conditions (linear mixed effect model: MH + ciprofloxacin: *p* < 0.005; M9 + tetracycline: *p* < 0.05). As such, the MIC of a mutant to a given secondary antibiotic generally did not correlate to its growth at sub-MICs of that antibiotic. Furthermore, the rank order of mutant fitness depended strongly on GxE interactions in sub-MICs of secondary antibiotics (aligned ranks transformation ANOVA: ciprofloxacin: *F*_22,108_ = 3.43, *p* < 0.001; d-cycloserine: *F*_22,108_ = 1.81, *p* < 0.05; tetracycline: *F*_22,108_ = 4.16, *p* < 0.001) (figure 4*b*). However, growth in the presence of sub-MIC of a secondary antibiotic was significantly correlated with growth in the absence of antibiotics in 7/9 tested conditions (electronic supplementary material, figure S3). In other words, fitness at sub-MICs of secondary antibiotic was not significantly related to the mutant tolerance of that antibiotic (i.e. its MIC), but was to its fitness in the absence of antibiotics. It is important to note that these correlations may be different if sub-MICs used were determined independently for each strain; however, in typical applications of antibiotics low concentrations are unlikely to be strain specific.

### (f) Pervasive pleiotropy as a possible source of complex genotype-by-environment interactions

In this study, we identified complex ways in which GxE interactions shape fitness of rifampicin-resistant mutants in the presence (figure 2*a*) and the absence of the primary drug (figure 2*b*), as well as their collateral effects (figure 3) and fitness in the presence of secondary antibiotics (figure 4). Why does fitness of these mutants and their rank order depend so highly and so unpredictably on the nutrient and antibiotic environment?

Like most known rifampicin resistance mutations [5], the 11 used in this study were point mutations in the *rpoB* gene coding for the β-subunit of RNA polymerase. Mutations in *rpoB* can alter global gene expression profiles, leading to some or even many genes being over- or under-expressed [42–45]. We explored whether such changes to global transcription levels were caused by the mutations we studied and if they were dependent on the nutrient environment, using three mutants (Q513L, Q513R and H526Y). We measured their transcriptome using *RNA-Seq* in LB and M9 and compared it to the expression levels observed in the WT strain in each respective medium.

**Figure 5.**
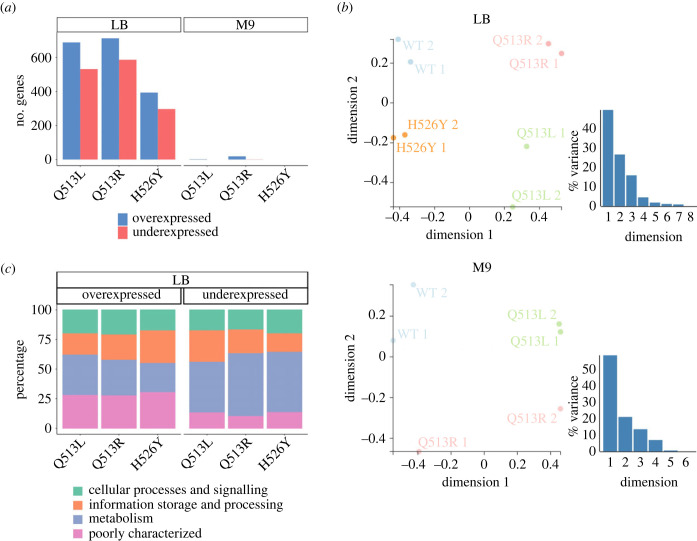
Pleiotropic effects of *rpoB* mutations on the transcriptome. (*a*) Number of differentially expressed genes of 3 *rpoB* mutants grown in LB (left) or M9 (middle), as compared to the WT grown in the same media. Threshold for differentially expressed genes was a false discovery rate of *p* < 0.05 and absolute log_2_ fold change of >1. (*b*) Multidimensional scaling (MDS) plots of normalized expression (CPM) values of all genes in both LB and M9. This plot visualizes the distance between samples based on the similarity of their gene expression profiles. H526Y in M9 is not shown as our analysis determined there were no differentially expressed genes. (*c*) Percentage of differentially expressed genes in LB divided into clusters of orthologous genes (COG) categories. We do not provide this analysis for M9 as the number of differentially expressed genes was very low.

The effects of rifampicin resistance mutations on the global transcriptome were often highly pleiotropic, significantly altering the expression levels of as many as several hundred genes (figure 5*a*). The sets of genes that were over- and under-expressed in each environment were mutant-specific, meaning that each rifampicin resistance mutation resulted in a unique transcriptional response (figure 5*b*). Similarly, the transcription profile of each mutant was different between nutrient environments (figure 5*a*). Together, these results show that the pleiotropic effects of rifampicin resistance mutations on gene expression levels are often large, mutant-specific and affected by the environment.

In some instances, changes to the expression levels of one or more specific genes can impact bacterial fitness in a given environment, by altering a key phenotype [43]. In general, we did not find such straightforward relationships between fitness measurements and individual key genes being over- or under-expressed. In one specific case (Q513R grown in LB), we found increased expression of the outer membrane porin gene *ompF. ompF* deletion has been implicated in increased resistance to a range of antibiotics including d-cycloserine, tetracycline and chloramphenicol [46]*.* Increasing the uptake of these antibiotics through upregulation of *ompF* could explain the increased sensitivity of this mutant to chloramphenicol and tetracycline (figure 3*a*). However, we observed no increased sensitivity to d-cycloserine, another substrate of this porin channel. Further, we found increased expression of *ompF* repressor *ompR* in H526Y grown in LB, but no concurrent decreases in sensitivity compared to the WT. We did not identify over- or under-expression of specific resistance genes to any of the secondary antibiotics used in this study. As such, the highly pleiotropic effects of *rpoB* mutations on transcriptomic profiles prevented us from drawing direct explanatory links as to how genotype-by-environment-by-antibiotic interactions shape bacterial fitness. Instead, it is more likely that the dependence of fitness effects on nutrient composition stems from the cumulative effect of altering expression levels of many genes, especially as many of the genes with altered expression levels are involved in metabolism (figure 5*c*).

## 3. Discussion

The global challenge of antimicrobial resistance necessitates novel strategies in addition to ongoing efforts to develop new drugs [47]. Understanding the evolutionary forces that shape the emergence and spread of resistance, especially multi-drug resistance, underpins such key strategies. While much work has been put into describing resistance evolution and the dynamics of resistant populations [48], much less is known about how those dynamics depend on GxE interactions [15,49,50]. In this study, we identified complex GxE interactions shaping the fitness effects of 11 highly pleiotropic *rpoB* mutations in 3 common laboratory nutrient environments, in the presence and absence of the primary antibiotic rifampicin or a secondary antibiotic with a different mechanism of action. The extent of fitness variation across environments was remarkably large. Perhaps most strikingly, we found that GxE interactions could alter the rank order of mutant fitness, while also affecting the direction and the strength of collateral effects against secondary antibiotics.

The fitness of a mutant in the presence of the selective antibiotic and in its absence plays a role in determining the evolutionary fate of a novel resistance mutation. As such, GxE interactions that alter mutant fitness between environments, and especially those that alter the rank order of fitness between mutants, result in more complex evolutionary dynamics. As one mutant can have a selective advantage (relative to other mutants) in one environment but a disadvantage in another, understanding and especially predicting the dynamics of resistance evolution becomes contingent on knowing fitness effects across a range of environments, not just in one. Though it has been previously observed that fitness in the presence or the absence of antibiotics can vary between environments [15,20,49,51], this factor is often omitted in studies characterizing resistance mutations and their costs [21,24,25,52,53]. Indeed, surveying a sample of 300 papers published in 2019 that performed antimicrobial susceptibility testing on *E. coli* isolates showed that only 1.5% assessed resistance in multiple nutrient environments. While a further 4% partially considered environmental effects by observing viability in both liquid and agar cultures, it is clear that the vast majority of resistance characterization occurs in only one nutrient context. For example, in a recent study examining the extensive collateral effects of a large number of antibiotic-resistant *Mycobacterium tuberculosis* strains (including several *rpoB* mutants), all MICs were tested in only a single nutrient environment [54]. Furthermore, MH media is often used as a ‘gold standard’ for MIC determinations owing to its stable pH and low concentrations of antibiotic inhibitors, and yet measurements of fitness and MIC in MH do not correlate to those made in LB or M9. Our study highlights how pervasive GxE interactions can be in shaping bacterial fitness, further emphasizing the need to experimentally measure fitness of resistant mutants across a range of environments. Nearly every surveyed paper referenced susceptibility testing guidelines published by either by CLSI or EUCAST to describe their methodology; as neither yet mentions the advantages of examining multiple growth environments, updating these guidelines could provide an avenue for the rapid adoption of more rigorous screening protocols.

Environmental variation influencing resistance profiles in such an unpredictable manner is particularly pertinent in clinical settings, where bacterial infections can colonize a variety of microenvironments and ecological niches around the human body [55–57]. Exploiting collateral sensitivity between antibiotics has been proposed as a novel therapeutic strategy that could slow down resistance evolution by exposing strains carrying resistance to one drug to a second antibiotic with an associated increase in sensitivity [12,58]. However, complex GxE interactions that alter the direction of collateral effects between nutrient environments [19], as well as different resistance mutations exhibiting different collateral sensitivity profiles [54,59–61], pose a difficulty for implementing such treatment strategies. While we tested laboratory media environments that are artificial and not necessarily representative of physiological growth environments, it is likely that such GxE interactions occur in more realistic environments as well. *Escherichia coli* is implicated in a wide range of infection sites including the urinary tract, bloodstream and gastrointestinal tract [62], all of which have unique microenvironments; our data suggest that resistant mutants may not necessarily exhibit the same collateral effects to secondary antibiotics in each of these environments. For example, data from fosfomycin-resistant *E. coli* strains (with a variety of different resistance alleles) show that variation of the microenvironment even within the same bodily site can alter the MIC of strains in a genotype-dependent manner [63]. Together with such previous studies, our data suggest that complex GxE effects on collateral effects might be common and complicate the design of resistance-proof therapeutic strategies.

The effects of rifampicin resistance mutations on global transcription levels likely underpin the pervasive GxE interactions we observed [10,21,44,45]. Indeed, the altered expression of 15–20 genes has been used to predict the levels of resistance to secondary stressors in evolved populations of *E. coli*, suggesting that altered expression of a few genes may be responsible for determining collateral effects [64]. Which exact genes are responsible for influencing the sensitivity to secondary drugs may depend on the antibiotic's mechanism of action or pharmacological properties. Specific mechanisms that might give rise to collateral effects include diverse cellular processes such as cell membrane charge, cell wall thickness, metabolic networks and SOS response [65,66]. This diversity, in turn, makes predictions of collateral effects challenging—difficulties which are exacerbated by the fact that different *rpoB* mutations affect the transcriptome in different (mutant-specific) ways [10,21]. Which mechanisms of resistance are associated with highly pleiotropic effects, and whether resistance that is not associated with pleiotropic effects on transcription can also give rise to complex GxE interactions on fitness and collateral effects, remain to be explored [19].

When studying the factors that shape antibiotic resistance evolution, most studies rely on characterizing the fitness of resistance mutations under a range of antibiotic and genetic conditions. However, much of these data are generated from experiments in a single laboratory medium. Similarly, resistance in clinical isolates is typically determined through growth on a single nutrient medium in the presence of a fixed dose of an antibiotic [67]. Our findings demonstrate pervasive GxE interactions that result in large variability in fitness of rifampicin-resistant mutants across different nutrient environments and their ability to tolerate secondary antibiotics. These effects, while likely linked to the pleiotropic effects of mutations in *rpoB* on global transcription, were unpredictable and seemingly stochastic. Together with previous studies that identified pervasive GxE effects on fitness of resistance mutants [15,16,19,50], our work points to the need for caution when extrapolating fitness estimates across environments and identifies the need to re-evaluate how we assess and evaluate resistance in experimental and clinical settings.

## 4. Materials and methods

### (a) Bacterial strain and culture conditions

*Escherichia coli* strain BW25113 Δ*tolC* was used in all experiments in this study. This strain is part of the Keio collection [68]. It should be noted that similar effects of environment and genotype on resistance profiles have been reported in strains with a functioning *tolC* [19], as well as in other species [61]. Strains were stored as 15% glycerol stocks at −80°C. Liquid cultures were grown at 37°C with shaking at 200 r.p.m. Agar plates were grown at 37°C without shaking. Culture media used were LB, MH and M9 supplemented with 2% glucose and 0.2% casamino acids.

### (b) Antibiotics

All antibiotics, their solvents and the range of concentrations tested can be found in electronic supplementary material, table S1.

### (c) Rifampicin-resistant mutant selection

*rpoB* mutants were generated with a fluctuation assay-based protocol in which mutations were first generated during growth in a non-selective environment before plating on selective agar plates to isolate resistant colonies. With this method, the likelihood of generating more than a single mutation is very low given the mutation rate. Firstly, overnight cultures were prepared by inoculating 1 ml MH from frozen stocks. The following morning, cultures were diluted 1 in 100 in 1 ml fresh MH media and grown for 24 h. Evolved cultures were spread on MH agar plates containing 20 µg ml^−1^ rifampicin and grown for a further 24 h. Colonies were picked and grown in liquid MH overnight for storage. Eleven unique *rpoB* mutations were generated, identified through Sanger sequencing of the *rpoB* gene. We carried out Illumina whole-genome sequencing on these strains to confirm the presence of only a single mutation (MicrobesNG performed genome sequencing then adapter trimming with Trimmomatic v 0.30 [69], with a sliding window quality cut-off of Q15). Trimmed reads have been deposited as FASTQ files to the US National Centre for Biotechnology Information (NCBI) Sequence Read Archive database under Bioproject accession number PRJNA988908.

### (d) Fitness measurements

Fitness measurements in figure 2 were obtained using a CLARIOstar Plus microplate reader. Overnight cultures were prepared in respective media (LB, MH or M9) from frozen stocks, before being diluted 1 in 1000 in the same media, with or without rifampicin at 8 µg ml^−1^. We used this concentration of rifampicin after measuring it as the MIC of the WT in MH using standard log_2_ dose–response experiments (see below). While some growth of the WT was observed in both LB and M9 at 8 µg ml^−1^ of rifampicin, suggesting that in these environments the MIC is higher, we wanted to keep the concentration of antibiotic consistent when comparing growth of resistant mutants so did not change it between environments. OD_600_ was measured every 10 min for 24 h at 37°C with shaking. Seven biological replicates were taken for each condition. Growth curve analysis was carried out using R and the package *growthcurver*. We used the empirical AUC between 0 and 24 h as our measurement of fitness, and values were normalized against the fitness of the WT in antibiotic-free MH. To test whether there was a significant change in rank order between nutrient environments in figure 2*a*,*b*, we performed an aligned ranks transformation ANOVA using the *art* function in the R package *ARTool*. In this model, we used AUC as the response variable, with nutrient environment, mutant (genotype) and the interaction between the two as explanatory variables. The WT was excluded from the analysis in figure 2*a* as its growth was greatly diminished in the presence of rifampicin. Tukey's pairwise comparisons were added to the analysis in figure 2*b* to determine which mutants had significantly different fitness from one another. The linear regression in figure 2*c* was performed with the *lm* function in R. Welch's two sample *t*-tests were used to test for significance between the fitness of specific pairs of mutants. To investigate the interaction between mutant, nutrient environment and the presence of rifampicin, a mixed effect model was fitted using the *aov* function with nested error terms of nutrient environment, rifampicin concentration and replicate.

Fitness measurements in figure 4 were obtained using a CLARIOstar Omega microplate reader with stacker. Overnight cultures were prepared as above, then diluted 1 in 1000 in media supplemented with a sub-MIC of antibiotic. The sub-MIC used for each antibiotic/media combination was determined by obtaining the MIC of every mutant in every antibiotic/media combination through standard dose–response experiments (see below), and then halving the lowest MIC of all mutants in that environment. For example, the lowest MIC of ciprofloxacin in MH media was 0.001 µg ml^−1^ (shared across several mutants), therefore the sub-MIC of ciprofloxacin used for all mutants in MH was 0.0005 µg ml^−1^. OD_600_ was measured every 30 min for 24 h at 37°C with shaking before each reading. Four biological replicates were measured for each condition. As described above, we used AUC as fitness and measurements were normalized to the fitness of the WT in antibiotic-free MH. To determine whether the fitness of a mutant at sub-MICs could be predicted from its MIC (figure 4*a*), we fitted a linear mixed effect model using the *lme* function from the R package *nlme*, with fitness (AUC) as the response variable, MIC as the fixed effect, and mutation (genotype) and replicate as random effects. To test whether there was a significant change in rank order of fitness between nutrient environments in the presence of sub-MICs of secondary antibiotics (figure 4*b*), we performed an aligned ranks transformation ANOVA as previously described.

### (e) Determining minimum inhibitory concentrations

Overnight cultures were prepared in respective media then diluted 1 in 100 in the same media supplemented with antibiotic at a range of concentrations (electronic supplementary material, table S1), prepared by serial dilution. Three biological replicates were tested. OD_600_ measurements were taken before and after 24 h of incubation. Blank values were subtracted from the final measurements, then MIC was determined as the lowest concentration at which at least two out of three replicates failed to reach an OD_600_ greater than 0.1. In more than 80% of cases this concentration was the same in all three replicates. The MIC of each mutant was normalized to the MIC of the WT in each respective antibiotic–media combination.

### (f) RNA-Seq

We conducted RNA-Seq analysis on three *rpoB* mutants to investigate their effect on the transcriptome (phenotype). We selected two strains with a mutation in the same residue (Q513L and Q513R) and a random third mutant (H526Y). RNA-Seq was carried out in two nutrient environments (either LB or M9) as the environments with the highest and lowest growth rates (averaged across all mutants). RNA was extracted using QIAGEN RNAprotect and RNEasy kits, according to manufacturer's instructions. Briefly, approximately 7.5 × 10^8^ RNAprotect stabilized cells harvested at OD_600_ = 0.7 were digested by incubating with 200µL TE (QIAGEN) containing 15 mg ml^−1^ Lysozyme (Thermo Fisher Scientific) and 10 µl Proteinase K (QIAGEN) for 10 min at room temperature. RNA was then purified using RNEasy spin columns. Samples were sent to Azenta Life Sciences (Germany) for quality control, rRNA depletion, library preparation and sequencing with the Standard RNA-Seq service using an Illumina NovaSeq platform and 150-bp paired end reads. Raw reads have been deposited in the NCBI Sequence Read Archive database under Bioproject accession number PRJNA988908.

Sequencing adapters and low-quality regions were trimmed using Trim Galore (v 0.6.7) (https://github.com/FelixKrueger/TrimGalore) using default parameters and reads shorter than 50 bp after trimming were discarded. rRNA reads were removed with BBDuk (v 38.18) (https://sourceforge.net/projects/bbmap) using the bundled rRNA k-mers database. Remaining mRNA reads were aligned to the reference genome (CP009273) using Bowtie2 (v 2.2.5) [70] and count tables were constructed from the resulting SAM files using featureCounts (v 2.0.1) [71]—see electronic supplementary material. DEGUST [72] was used to perform differential expression analysis using the voom/limma method with a false discovery rate threshold of *p* < 0.05 and absolute log fold change of >1, and to generate multidimensional scaling (MDS) plots. To determine COG categories for differentially expressed genes, coding sequences of the reference genome were downloaded from RefSeq and functionally annotated using the online instance of eggNOG-mapper (http://eggnog-mapper.embl.de/) with default parameters and Diamond as the underlying alignment algorithm [73–75].

### (g) Antimicrobial susceptibility testing literature review

The literature survey examining antimicrobial susceptibility testing in multiple environments was conducted using PubMed's database. The search terms were ‘antibiotic-resistant’, ‘isolate’ and ‘Escherichia coli’; filters were used to exclude review publications and select only those published in 2019, providing a current assessment without bias from COVID-19. The first 300 of 1351 results were annotated according to the distinct environments in which they measured antimicrobial susceptibility or MIC. In cases where the used growth medium was unstated, it was assumed that measurements were taken only in one environment.

## Data Availability

The data are provided in electronic supplementary material [76].
